# Impact of the association of strength training with neuromuscular electrostimulation on the functionality of individuals with functional decline during senescence: A systematic review and meta-analysis

**DOI:** 10.1016/j.clinsp.2025.100586

**Published:** 2025-02-07

**Authors:** Dhianey de Almeida Neves, Leonardo Costa Pereira, Kerolyn Ramos Garcia, Frederico Santos de Santana, Rhenan Yoshio de Caldas Fujita, Beatriz dos Santos Faria, José Antônio Alves de Oliveira, Carlos James Zeidan Silva Filho, Margô Gomes de Oliveira Karnikowski

**Affiliations:** a Faculdade de Ceilândia, Universidade de Brasília (UNB), Brasília, DF, Brazil; b Centro Universitário Euro-Americano (UNIEURO), Brasília, DF, Brazil

**Keywords:** Resistance training, TENS, Functional physical performance

## Abstract

**Introduction:**

One of the parameters observed in functional capacity over the years is the decrease in neuromuscular responses, a fact that is attributed to the contemporary lifestyle. Thus, there is a need to carry out interventions that induce the improvement of functional capacity. Some studies have associated electrostimulation (NMES) with Strength Training (ST) to enhance the results in improving neuromuscular function. However, little is known about the effects of this association due to the numerous protocols to be manipulated. Furthermore, adaptive responses to strength training are dependent on volume and intensity manipulation.

**Objective:**

To investigate the influence of ST, concomitant with NMES (NMES+) on functional capacity.

**Methods:**

This is a systematic review with meta-analysis. For the search of the articles, descriptors associated with functional capacity and NMES+ were selected in the Cochrane, PubMed, Embase and VHL meta-searcher databases. Inclusion criteria were articles that presented neuromuscular electrostimulation superimposed on voluntary contraction and ST intensity control; and that did not have a therapeutic purpose. The analysis of titles, abstracts and data extraction were performed by trios of reviewers. To assess the qualities of scientific evidence, the risk of bias was analyzed through the ROB2 tool, meta- analysis and evaluation of the quality of evidence (GRADE).

**Results:**

This meta-analysis selected 3 studies. The main outcomes observed in the studies were agility, balance, cardiorespiratory capacity and strength and power. A significant improvement in effect estimates for cardiorespiratory capacity alone was observed between the two studies.

**Conclusion:**

Despite the significant effect of the use of NMES+, in relation to ST in isolation, the quality of the evidence was considered low, probably due to the limited number of scientific evidence found, requiring further studies to identify the real effect of this association.

## Highlights

• NMES+ can improve the aerobic capacity of individuals in functional decline.
• NMES+ appears to improve physical functional capacity in general.
• More RCTs are needed to better evaluate the evidence found.

## Introduction

The improvement of health and quality of life are directly related to the competence to idealize and perform the essential activities of daily life autonomously and independently, thus entitled to functional capacity.^1^ Therefore, the modern lifestyle, associated with the process of senescence, induces reductions and impairment of these capacities.^2^^,^^3^ Individuals' physical and cognitive abilities often undergo changes in various systems as they age, which can influence biopsychosocial changes.^4^^,^^5^ These changes vary greatly from person to person but generally involve a gradual decline in various physical and cognitive functions.^6^^,^^7^ Therefore, interventions that induce the improvement of functionality are necessary.

One of the parameters observed in functional capacity during senescence is the decrease in neuromuscular responses.^8^ The literature shows that physical exercises, in a systematic way, with volume, intensity, and range of motion control, among others, can induce neuromuscular adaptations and body composition, capable of minimizing the deleterious effects of age on functionality.^9^, ^10^, ^11^

Among the various modalities of physical exercises, strength training stands out, capable of generating adaptations in both morphological and physiological parameters, linked to the musculoskeletal system^12^ and energy metabolism,^13^ in a safe and efficient way.^14^ These changes come from voluntary contractile stimuli. Of the various strategies for manipulating or increasing strength training methods, another tool called electrostimulation^15^ is observed, which seems to complement gaps not filled by the traditional strength training method, especially with regard to the differentiated recruitment of the various types of muscle fiber through the various types of electrical currents.^16^

Neuromuscular Electrostimulation (NMES) consists of the application of low, medium, and high-frequency electrical stimuli through electrodes, located on the muscle surface, or at the motor point of the muscles.^17^ When stimulated, there are activations in the intramuscular branches of the motoneurons in the muscle fiber bundles, thus generating involuntary contractions.^18^ Currently, it has been constantly used for therapeutic purposes in muscle rehabilitation and to improve performance.^19^, ^20^, ^21^

Intuitively, some studies have associated electrostimulation with strength training to enhance the results in improving neuromuscular function.^19^^,^^22^^,^^23^ However, the effects of this association on functional physical capacity and strength in old individuals are still unclear. In addition, most of the reviews carried out with this population do not include only studies that controlled the load of strength training.^24^, ^25^, ^26^ Taking into account that the results presented are dependent on the dose and response to training.

Having presented the arguments, this group estimates that, old individuals that make use of neuromuscular electrostimulation associated with strength training, would have a greater effectiveness in strength and functional physical capacity, compared to old individuals that make only isolated strength training. Thus, according to the arguments presented, the present review intends to analyze the effects of strength training concomitant to neuromuscular electrostimulation, on muscle strength and functional physical capacity of old individuals, through Systematic Review and Meta-analysis.

## Methods

### Experimental design

This research is a systematic review article with the presence of analysis, which followed all the guidelines proposed by the Cochrane Database of Systematic Reviews, together with the guidelines described by the PRISMA group.^27^ In this perspective, in order to discern information about the test modalities in the evaluation of strength and functional capacity responses in strength training concomitant to electrostimulation, the authors followed the guidance of starting from a guiding question, which is: Do old people submit to strength training associated with neuromuscular electrostimulation obtain greater gains in strength and functional physical capacity, compared to old people who perform only strength training?

The study was careful to verify in the scientific literature, through the PROSPERO database and the Cochrane Database of Systematic Reviews, on the CRD registration 42,023,408,049, the existence of similar works in order to resolve the need for a new review. Specific descriptors were searched using the same algorithms used in the review, however, no studies identical to the one proposed by the present study were found.

Articles related to electrostimulation and strength training were analyzed in the following databases: Cochrane, PubMed, Embase, and the VHL meta-searcher. The present research divided its elaboration into stages. The first stage was to carry out a literature review, with a random and empirical search on the proposed theme. In the second stage, some search descriptors were chosen in the Embase and PubMed databases. In the third stage, systematic reviews were searched in the Cochrane Library, in order to identify gaps in the theme and new keywords. The searches were carried out using the search algorithms using the Match Terms selected between 28/03/2023 and 25/04/2023, and their combination is presented by algorithms exposed in the supplementary documents of this work.

### Peer review

After selecting and inserting the algorithms for searching and removing duplicates with the help of the Mendeley Desktop software, two trios of reviewers read the titles and abstracts of the articles, based on: The inclusion of articles that 1) Presented a chronic intervention of strength exercises; 2) With protocols that have a minimum duration of two weeks; 3) With neuromuscular electrostimulation superimposed on voluntary contraction; 4) Only complete works were included in this study; 5) Identified in the databases already mentioned in this work; 6) Non-duplicated articles; 7) That did not present a therapeutic purpose; 8) Original articles that manipulated a sample with human beings; 9) That did not present a sample composed of individuals with musculoskeletal changes in the joints, behavioral and neurological alterations, which compromised the contractile and/or metabolic capacity. The exclusion criteria were: 1) Absence of functional tests and/or their appropriate metrics; 2) Experimental design unable to evaluate the concomitant effect of neuromuscular electrostimulation superimposed on ST; 3) Mean age <50 years; 4) No control of ST intensity.

After analyzing the criteria and reading the full texts, two trios of reviewers were responsible for discussing the inclusion of articles for final analysis. Articles that disagreed on inclusion were analyzed during the meetings scheduled by a fourth reviewer. Data were extracted through the observations of peer reviewers independently. After the analyses, a third reviewer was responsible for mediating the disagreements between the selected data.

### Outcomes

#### Types of studies

Intervention studies in which the investigator manipulates the variables to obtain a cause-and-effect relationship between the expected outcomes. Randomized Clinical Trials (RCTs) or studies that have a quantitative, experimental, quasi-experimental character, randomized or not, containing intervention of concomitant use between electrostimulation and strength training with chronic characteristics, with or without the presence of a control group.

#### Types of participants

All studies included in this research recorded the age of their participants over 50 years old, without age limits, and of both sexes. Thus, the study aimed to cover the different legislations of underdeveloped, developing, and developed countries, as presented by the World Health Organization (WHO).^28^ Whether or not they have experience with strength training, with various physical fitness conditions^29^ for neuromuscular^30^ and cardiometabolic^31^ parameters.

#### Types of intervention

RCTs that detailed strength training interventions with various protocols as long as they presented the form of control of the variables of intensity, volume, frequency, and duration of the protocol were included; These interventions should also present the specifications of the form of current used to carry out the electrostimulation protocol, specifying: the pulse frequency, being defined as up to 50hz, as low frequency, 50hz up to 1000hz medium frequency, and high frequency above 1000hz, which may occur by Pulse Burt.^18^^,^^32^ Intervention protocols must be concomitant with electrostimulation. All interventions should respect universal ethical precepts of the Declaration of Helsinki and Resolution 196/96 of the National Health Council and International Ethical Guidelines for Biomedical Research Involving Human Subjects (CIOMS/OMS 1982 and 1993) and the International Guidelines for Ethical Review of Epidemiological Studies (CIOMS, 1991).

#### Types of outcome measures

The RCTs that make up the results of this research should use different measures to assess the results relevant to functional capacity. It can generate a variety of rating scales, present pre- and post-intervention numerical data, and/or comparisons between different intervention or control groups. For each outcome of interest, the authors therefore tried to list the most common and relevant measures or tools related to assessments of neuromuscular physical capacities. These studies should not present participants who used their intervention as a cure process for any pre-existing disease.

### Analysis of meta-analysis

Detailed statistics were performed on the RCT data using Medcal 20.0 software, calculating effect size, by Cohen *d*, confidence intervals and p-values relevant to the research question. Funnel and forest plot graphs were generated to visualize the distribution of the data and highlight the individual results of each study. A meta-analysis was applied to combine these results and calculate the overall effect of the intervention, and then the heterogeneity between the studies was assessed using statistics such as the Cochran's Q test and the I^2^ index, allowing us to explore the variability between the studies before proceeding with the interpretation of the results. Every meta-analysis was performed using the robust random effects of the variance model. The effects were considered statistically significant with a p-value ≤ 0.05.

### GRADE method for stratification of the level of evidence

The quality of evidence was assessed using the Grading of Recommendations Assessment, Developing and Evaluation (GRADE)^33^ method, which evaluates evidence at four levels: high, moderate, low, or very low. The quality of the evidence reflects confidence in the results and is classified with factors that decrease the quality of the evidence, such as:

#### Risk of bias

To assess the risk of bias, a Cochrane risk-of-bias tool for randomized trials (RoB 2)^34^ was used, which provides a systematic way to organize and present the evidence found related to the risk of bias, evaluating five domains related to bias in the randomization process, deviations from the intended intervention, bias due to missing data, bias in measuring outcomes and bias in reporting outcomes. Some questions were established for the judgment of the risk of bias, which could be considered low risk of bias, some suspicions, or high risk of bias.

#### Inconsistency

Assessed by analyzing the variation of effect estimates (point estimates); overlap of 95 % CIs; statistically significant heterogeneity (*p* < 0.05); and significant I^2^ value, without explanation of the factors that may change it.

#### Indirect evidence

The analysis of indirect evidence related to the internal and external validity of the studies in relation to the outcomes that answer the research question, directly or indirectly, the more indirect the evidence, the more levels the study loses.

#### Imprecision

Inaccuracy was assessed through the total number of participants and the analysis of confidence intervals. If the confidence intervals in the effect analyses touch the center line, with no significant result, it can be lowered due to inaccuracy by one level, if it completely exceeds the center line, obtaining the point estimate of the opposite side of the expected effect, it can be lowered by two levels.

#### Bias of publication

Assessed through the analysis of the search strategies of the studies, relating the main databases used and the presence of conflicts of interest in relation to industry financing. The funnel graphs were presented to verify the distribution of the studies and sensitivity test (Egger's). Each of these classifications can reduce the quality of evidence by a level or two, depending on each outcome.

## Results

**Fig. 1:**
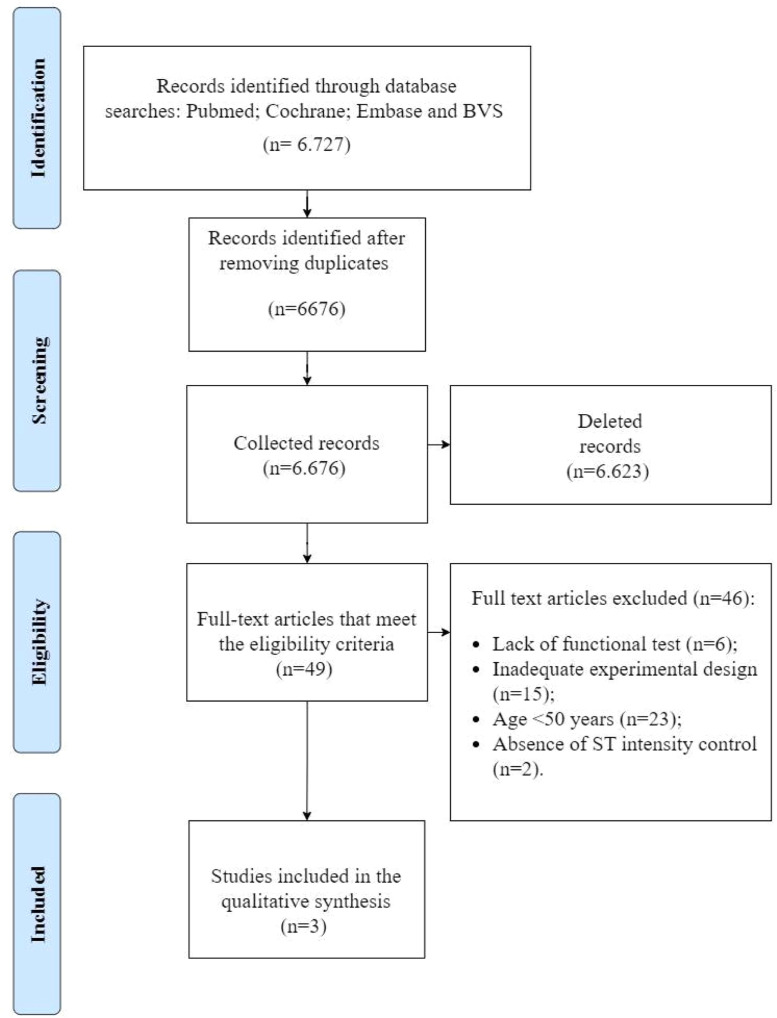
Flowchart of the process of analysis, eligibility, exclusion and inclusion of studies in the systematic review.

This meta-analysis assessed three RCTs with similar interventions, relating strength training with electrostimulation to the improvement of strength and functional physical capacity in individuals aged over 55-years. The flowchart with the selection of studies is shown in Fig. 1.

After the insertion of the selected algorithms for the search strategies in the databases, a total of 6727 studies were initially located, only 49 studies with full texts were included as potentially eligible. In the stage of article selection, after reading the full texts and pre-established discussions with a fourth reviewer, 3 studies^35^, ^36^, ^37^ were selected.

Regarding sociodemographic data, the studies covered a total of 155 individuals, aged between 55 and 96 years. One study included participants of both sexes^35^ and the other two included only women.^36^^,^^37^ All included studies were conducted in the European Continent (Spain). The level of conditioning of the participants was not reported in one study,^35^ being identified as inactive in the other two.^36^^,^^37^

Regarding the exposure of the participants, only one study made comparisons with varied interventions and an inactive control group,^35^ however, the analyses were made only with the RT and NMES+ interventions. The other two studies had an active control group (ST)^36^^,^^37^ (Table 1). All selected studies performed a training volume above 10 weeks, with isotonic muscle contractions and strength training intensity at 40 % of 1RM.^35^, ^36^, ^37^ The weekly training frequency was three^35^ times a week in one study and twice a week in the other.^36^^,^^37^ In addition, it was observed that all studies performed exercises for lower limbs, however, only two studies performed exercises for upper limbs.^36^^,^^37^

**Table 1:** Data regarding exposure of samples in each study.

	Sample			Exhibition				
	n	Sex	Age	Intervention				Control
				Exercise	% Load	Frequency (Hz)	Type	
**Caballer et al., 2014**	89	Male and Female	RT, *n* = 22, age = 85.5 ± 4.7-years; NMES+, *n* = 22, age = 83.6 ± 3.6-years; NMES, *n* = 22, age = 82.9 ± 4.3-years; CG, *n* = 23, age = 83.6 ± 5.6-years	Knee extension exercises	40 % / 1MR	50	NMES	RT
**Rodriguez et al., 2020**	34	Female	RT, *n* = 17, age = 59.7 ± 3.82-years; WB EMS+, *n* = 17, age = 63.1 ± 3.42-years	Squats, deadlifts and bench presses	40 % / 1MR	7 to 55	WB-EMS	RT
**Rodriguez et al., 2020**	32	Female	RT, *n* = 16, age = 59.71 ± 3.82-years; WB EMS+, *n* = 16, age = 63.06 ± 3.42-years	Squats, deadlifts and bench presses	40 % / 1M	7 to 55	WB-EMS	RT

RT, Resistance Training; CG, Control Group; NMES, Neuromuscular electrostimulation; NMES+, Neuromuscular electrostimulation concomitant with Resistance Training; MR, Maximum Repetition; WB-EMS, Whole body electrostimulation.

The electrostimulation used in the studies was low frequency, between 50 and 55 Hz. However, one study used two-pole surface electrodes,^35^ the other two used whole-body electrostimulation.^36^^,^^37^ The intensity of the electric current during training was measured from 150 to 350 milliseconds in two studies^36^^,^^37^ and up to the pain threshold in another study.^35^

The main outcome variables related to functional physical capacity used in most studies were balance,^35^^,^^37^ followed by strength and power,^35^, ^36^, ^37^ aerobic capacity^35^^,^^37^ and mobility.^35^^,^^37^ Most of the positive and significant results, when compared to the different moments assessed were manifested in the NMES+ protocols, however, the RT protocols also showed significant results, especially for strength gains. However, the studies reported no differences between the different intervention groups. Only the variables aerobic capacity, mobility, and strength showed differences (Table 2).

**Table 2:** Main outcome variables related in each study.

	Outcome	Functional capacity			
		CG	RT	NMES+	NMES
**Caballer et al., 2014**	Balance	−1.72	3.53	5.21	2.09
	Strength and power	−9.96^a^	−0.31^a^	−0.89^a^	1.44^a^
	Aerobic capacity	−4.42	2.87	2.30	4.58
	Mobility	24.84	−15.68	−19.92^a^	−7.38
**Rodriguez et al., 2020**	Balance	‒	7.60	−0.21	‒
	Strength and power	‒	39.39^a^	64.37^a^	‒
	Aerobic capacity	‒	4.47	27.68^a^	‒
	Mobility	‒	−0.22	−22.26^a^	‒
**Rodriguez et al., 2020**	Strength and power	‒	1.83	65.86^a^	‒

Significant differences of *p* < 0.05 between the pre- and post-intervention moments of the variables related to functional capabilities in each study (^a^).

### Risk of bias within studies

**Fig. 2:**
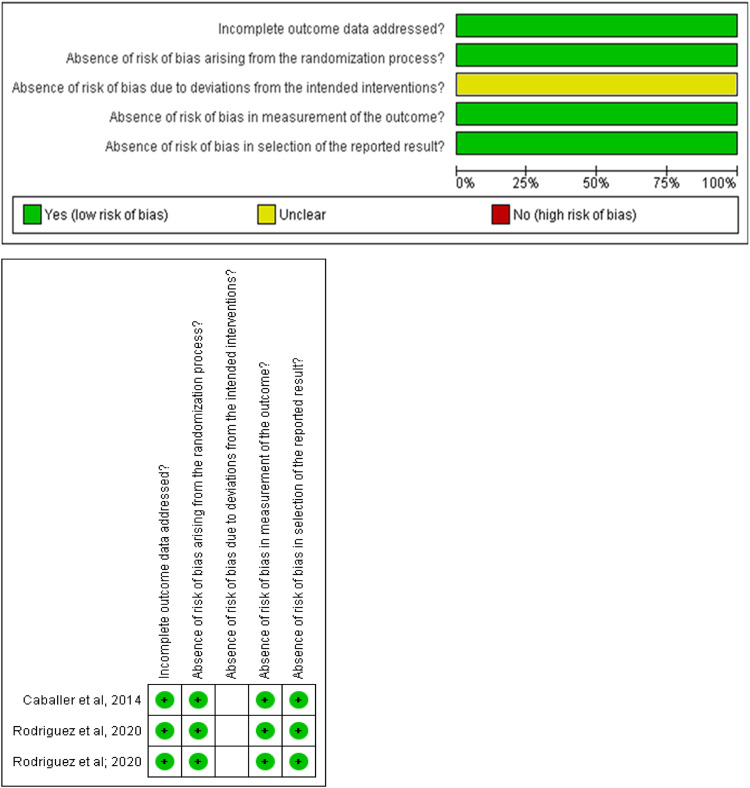
Analysis of risk of bias between studies.

Of the three included RCTs, 100 % were considered to have a low risk of bias, however, they had some concerns in the second domain, the risk of bias due to deviations from the intended interventions, in all studies (Fig. 2).

### Meta-analysis

**Fig. 3:**
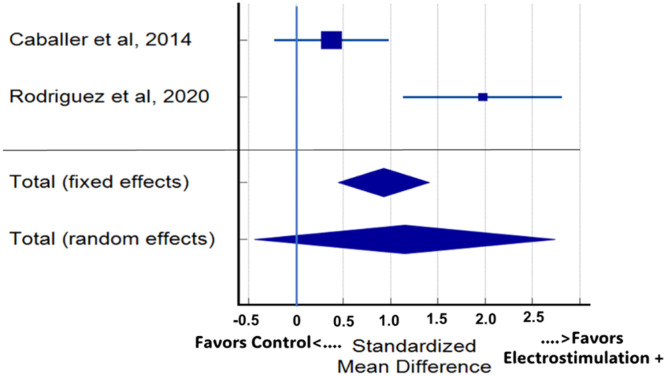
Meta-analysis for the variable aerobic capacity between the two studies.

**Fig. 4:**
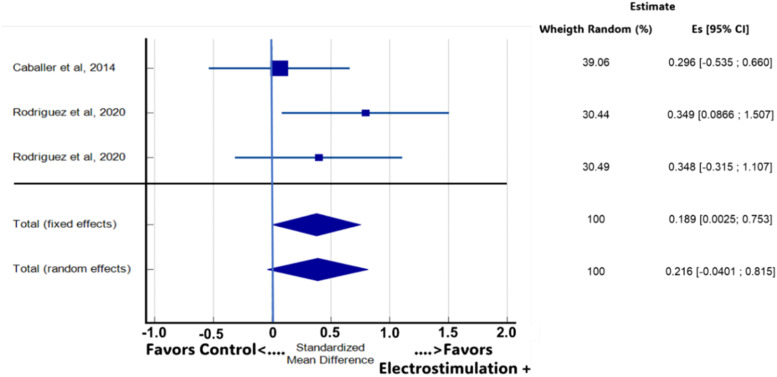
The Forest Plot graph of the meta-analysis shows the size of the effects of strength training superimposed on electrostimulation. The X axis indicates Cohen's *d* (ES), while the whiskers demonstrate the 95 % Confidence Interval for each study.

The result of the meta-analysis showed that in comparison with ST, in the four outcome variables related to functional physical capacity, NMES+ presented a significant result only for the aerobic capacity variable, in two studies^35^^,^^37^ (Fig. 3). The other variables, despite presenting an effect trend, did not present significant differences. When considering all studies and the means of the available outcome variables, the meta-analysis on the effects of electrostimulation superimposed on strength training in improving the functional capacity of the old people did not indicate a significant difference between the training conditions (*p* = 0.049; Es = 0.189; 95 % CI: 0.00247 to 0.753; Fig. 4).

**Fig. 5:**
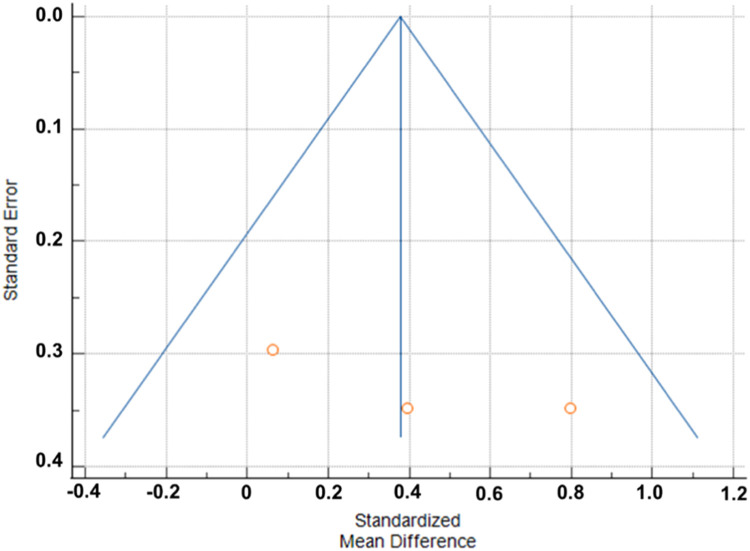
Funnel chart to the overall average of the variables related to functional physical capacity.

The quality of evidence for NMES+ compared to ST alone is summarized in Table 3. All the variables analyzed were classified as low or very low quality. The reduction in the quality of evidence is associated with variations in effect estimates; 95 % CI overlap; and statistically significant heterogeneity; I^2^ (inconsistency) 80.89 % for the balance variable and 62.73 % for the agility variable (two studies). The data show no significant effects, with the confidence interval crossing the no-effect line. As for the imprecision of the outcomes, the number of studies^35^, ^36^, ^37^ was insufficient. Regarding the result of aerobic capacity, the data indicate a small inconsistency in the variation of the effect estimates, a significant heterogeneity, with I^2^ of 89.84 %. For strength and power, inconsistencies were found in the effect estimates and the absence of statistical differences 95 % Cl. The funnel graphs adjusted by comparison of the meta-analysis for results showed asymmetry in the variables balance, aerobic capacity, and agility, with a significant Egger's test (*p* < 0.05), which suggested a possible publication bias. However, the N of studies is very low, and it is not possible to state (Supplementary Material), being evaluated only through the conflicts of interest of each study and the search strategies. For the means of the variables in general, the funnel graph is shown in Fig. 5, Begg's test (*p* = 0.1172) and Egger's test (*p* = 0.2756) indicating no publication bias.

**Table 3:** Meta-analysis (95 % CI) of the main outcome variables for each study and assessment of the quality of evidence (GRADE).

Outcome	Author	n	Quality of evidence (GRADE)	Weight	Meta-analysis (95 % Crl)
Balance	Caballer et al., 2014	22	⨁◯◯◯ Very low	58.79	(−0.487 to 0.710)
	Rodriguez et al., 2020	17		41.21	(−1.665 to −0.223)
Aerobic capacity	Caballer et al., 2014	22	◯◯ Low	65.49	(−0.225 to 0.981)
	Rodriguez et al., 2020	17		34.51	(1.135 to 2.812)
Mobility	Caballer et al., 2014	22	⨁◯◯◯ Very low	58.25	(−0.684 to 0.512)
	Rodriguez et al., 2020	17		41.75	(−1.550 to −0.124)
Strength	Caballer et al., 2014	22	⨁⨁◯◯ Low	40.2	(−0.499 to 0.697)
	Rodriguez et al., 2020	17		30.69	(−0.251 to 1.130)
	Rodriguez et al., 2020	16		29.11	(−0.315 to 1.107)

## Discussion

After the analyses carried out in the literature, no other review was identified that has a similar objective to the present review, being to analyze the effects of strength training concomitant to neuromuscular electrostimulation, on muscle strength and functional physical capacity of old individuals, through a Systematic Review with Meta-Analysis.

The studies selected in this review^35^, ^36^, ^37^ presented protocol conditions capable of verifying the effect of electrostimulation concomitant to strength training on adaptive responses related to the gain of functional capacity. However, only the outcomes strength and power, agility, and aerobic capacity showed superior results in the association of the two trainings. However, despite favorable gains to NMES+ training, only aerobic capacity showed statistical differences for effect estimates.

Some evidence indicates that the use of electrostimulation alone to improve aerobic capacity in sedentary individuals seems to induce significant adaptations.^38^, ^39^, ^40^ The results found in the present study seem to collaborate with this evidence. Nevertheless, when comparing NMES+ protocols with ST in isolation and their significant improvements in effect estimates, it is necessary to highlight the low power of evidence due to inconsistencies in the meta- analysis related to heterogeneity and inaccuracies associated with the N sample of the selected studies. This inconsistency may have occurred due to the manipulation of the outcomes in each study. Of the two studies^35^^,^^37^ that evaluated aerobic capacity, only one^37^ performed specific exercises for aerobic capacity, and the other^35^ performed only one training for lower limbs.

Despite the statistical differences found in some variables and the observed effect trends, the results in some studies^35^^,^^37^ were controversial, especially in relation to the strength and power variable, which in one study^35^ showed negative results for all groups. This result may be related to the age of the participants, which ranged from 55 to 96 years. The greater the age, the greater the decline in muscle mass and strength.^41^ Although the stimulation of exercise contributes to increased strength and muscle mass, the evidence is still unclear when the authors relate stimuli in an organism in functional decline.^42^^,^^43^ In addition, the study,^35^ which showed a decline in the strength variable, evaluated only the upper limbs through the handgrip test. However, it induced stimuli only in the lower limbs, so the authors can infer that the lack of specific stimuli in the upper limbs may have contributed to the reduction of strength in all groups, taking into account the principle of specificity.^44^ In addition, the negative result related to one of the studies may have contributed to the inconsistency in the results of the meta-analysis and consequently influenced the effect estimates, which had a positive trend for the NMES+ training group, but no significant differences were identified.

The adaptations generated for the development of strength are dependent on the combination of morphological and neural factors, such as the area and architecture of the muscle cross-section, muscle-tendon stiffness, recruitment of motor units, speed coding, synchronization of motor units, and neuromuscular inhibition.^45^ Therefore, it is necessary to induce loads that recruit a greater number of motor units and an adequate execution speed, to enhance strength gains.^46^ However, the intensities used in all studies were below 40 % of 1RM. It was not reported in the runtime control studies. However, a study carried out with young people compared the use of NMES+ to strength training, with load variations between 50 % and 80 % of 1RM, and observed no statistical difference between groups.^47^

The use of NMES+ results in decreased postural oscillation and greater stability of the ankle joint and improved balance in old individuals.^48^ However, the studies selected in this meta-analysis did not identify statistical differences between the groups and moments of each intervention, in addition, the effect estimates did not show significant differences, nor an effect trend, on the contrary, the effects were found completely across the non-effect line. Although the authors did not identify significant differences, we should point out that the quality of the evidence was considered very low, and the small sample size of the studies contributed to some inaccuracies in the results.

The agility outcome also presented a very low quality of evidence; however, the effect estimates had a positive trend, although they did not present significant results. In addition, the comparisons between the pre- and post-intervention moments of all studies for the NMES+ group showed significant differences in this outcome.

### Limitations of the review

This meta-analysis has limitations regarding the non-treatment of missing data, which may have influenced some outcomes and limited the analyses. Second, the authors did not have access to Supplementary Materials from some studies, which potentially met the eligibility criteria, because they analyzed some variables indirectly, reducing the number of studies selected. Third, it was not possible to perform meta-regression calculations, which may have influenced the sensitivity test of the studies and consequently the quality of the evidence. Fourth, the analyses of publication bias were inconclusive due to the low number of selected studies, although some tests (Egger's) were significant. Fifth, the search for studies was limited only to the databases mentioned, and studies were not identified through other databases or different methods.

## Conclusion

The results found in the present study show a greater advantage of using NMES+, compared to ST alone, for low-intensity exercises up to 40 % of 1RM, especially for the variable cardiorespiratory capacity. However, it cannot be said that NMES+ would be more effective in improving functional capacity in individuals in functional decline, mainly due to the scarcity of evidence and the low quality of the few evidences that were found, requiring the elaboration of new RCTs, which perform interventions with greater methodological rigor, with different training intensities, different types of electric current and a better adequacy of stimuli, more specifically for each outcome variable evaluated.

## Contributions of the study to clinical practice

The findings identified in this review present evidence related to the safety and efficiency of using NMES+ST, helping each professional with regard to prescription parameters and the positive prognosis in relation to reducing training loads, and preserving the joint integrity of the elderly, whether compromised or not., without compromising strength gains and strength improvements in this population.

## Authors’ contributions

Literature search: Dhianey de Almeida Neves.

Data collection: Dhianey de Almeida Neves, Carlos James Zeidan Silva Filho, Beatriz dos Santos Faria, Kerolyn Ramos Garcia, José Alves, Rhenan Yoshio De Caldas Fujita and Leonardo Costa Pereira.

Study design: Dhianey de Almeida Neves, Leonardo Costa Pereira, Margô Gomes de Oliveira Karnikowski, Frederico Santos Santana, Carlos James Zeidan Silva Filho, Beatriz dos Santos Faria, Kerolyn Ramos Garcia, José Alves and Rhenan Yoshio De Caldas Fujita.

Analysis of data: Dhianey de Almeida Neves, Leonardo Costa Pereira, Beatriz dos Santos Faria, Rhenan Yoshio De Caldas Fujita and Margô Gomes de Oliveira Karnikowski.

Preparation of the manuscript: Dhianey de Almeida Neves.

Review of the manuscript: Margô Gomes de Oliveira Karnikowski, Frederico Santos Santana and Leonardo Costa Pereira.

## Funding

This research did not receive any specific grant from funding agencies in the public, commercial, or non-profit sectors.

## Declaration of competing interest

The authors declare no conflicts of interest.

## Appendix Supplementary materials

Image, application 1
