# Supplementary material for: Impact of the association of strength training with neuromuscular electrostimulation on the functionality of individuals with functional decline during senescence: A systematic review and meta-analysis
Source: Clinics (Sao Paulo). 2025 Feb 7;80:100586. doi: 10.1016/j.clinsp.2025.100586 (PMC11850139; doi:10.1016/j.clinsp.2025.100586)
Supplement: Supplementary file 1 [file mmc1.docx]

**CLINIC-D-23-00595_ Supplementary Material**


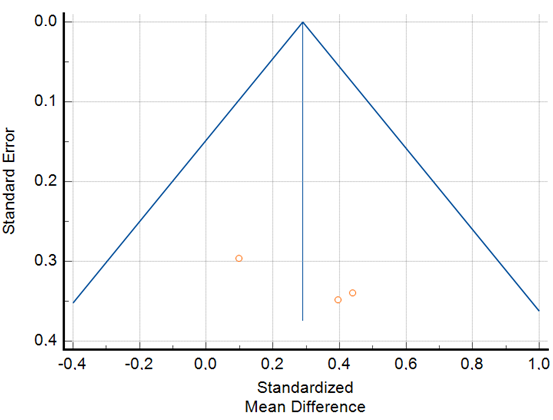


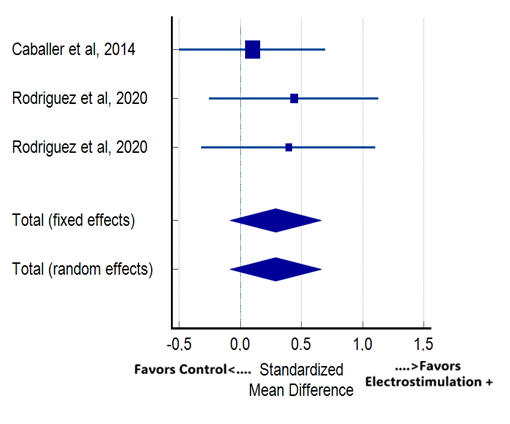


Effect size and funnel chart for strength and power.


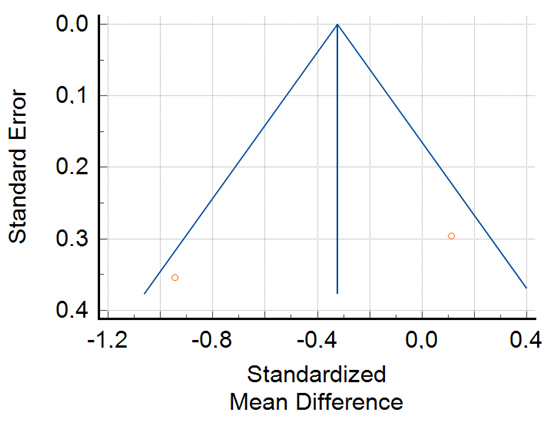


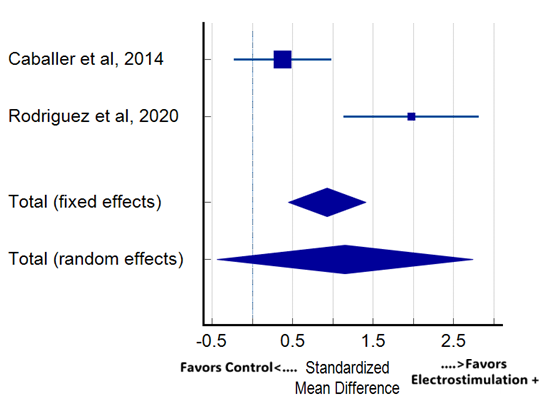


Effect size and funnel chart for Six walk test


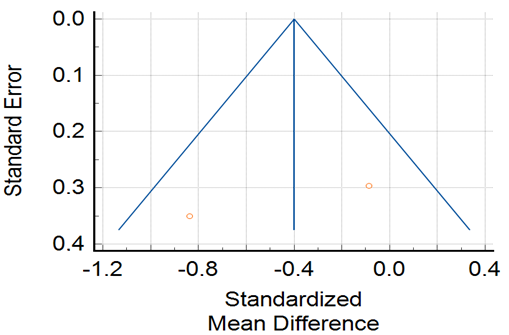


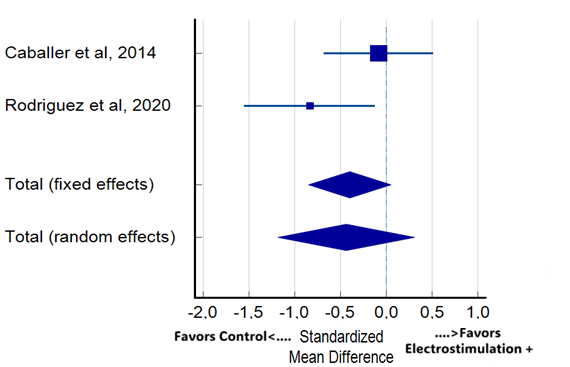


Effect size and funnel chart for tug


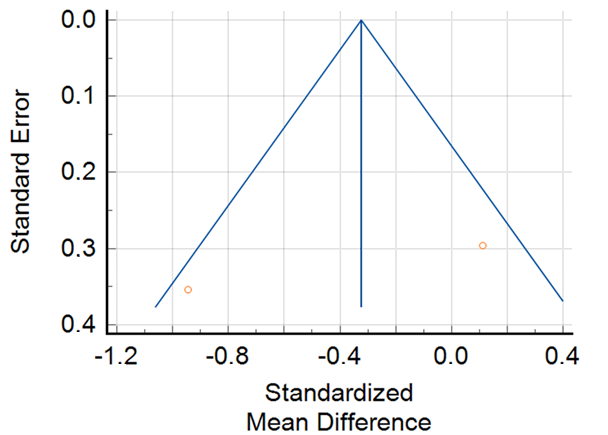


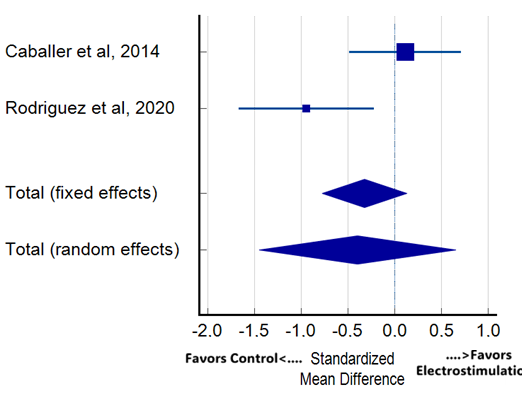


Effect size and funnel chart for balance.

**Author(s):** Dhianey de Almeida Neves; Leonardo Costa Pereira; Margo Gomes de Oliveira Karnikowski

**Question:** Strength training associated with electrical stimulation compared to isolated strength training for improvement of strength and functional capacity in the elderly

**Setting:** Would elderly people undergoing strength training associated with neuromuscular electrical stimulation show a greater gain in strength and functional capacity compared to elderly people who perform strength training alone?

**Bibliography:**

| **Certainty assessment** | | | | | | | **№ of patients** | | **Effect** | | **Certainty** | **Importance** |
| --- | --- | --- | --- | --- | --- | --- | --- | --- | --- | --- | --- | --- |
| **№ of studies** | **Study design** | **Risk of bias** | **Inconsistency** | **Indirectness** | **Imprecision** | **Other considerations** | **strength training associated with electrical stimulation** | **isolated strength training** | **Relative (95% CI)** | **Absolute (95% CI)** |  |  |
| **Balance (follow-up: range 4 weeks to 16 weeks; assessed with: Berg Balance Scale ; Scale from: -0,776 to 0,129)** | | | | | | | | | | | | |
| 2 | randomized trials | not serious | very serious^a^ | not serious | extremely serious^b^ | none | 39 | 39 | - | SMD **0.324 SD lower** (0.776 lower to 0.129 higher) | ⨁◯◯◯ Very low |  |
| **Agility (follow-up: range 4 weeks to 16 weeks; assessed with: Timed Up and Go Test )** | | | | | | | | | | | | |
| 2 | randomized trials | not serious | very serious^c^ | not serious | very serious^d^ | none | 39 | 39 | - | SMD **0.399 SD lower** (0.85 lower to 0.051 higher) | ⨁◯◯◯ Very low |  |
| **Aerobic capacity (follow-up: range 4 weeks to 16 weeks; assessed with: The 6-minute walk test )** | | | | | | | | | | | | |
| 2 | randomized trials | not serious | serious^e^ | not serious | serious^f^ | none | 39 | 39 | - | SMD **0.928 SD higher** (0.447 higher to 1.41 higher) | ⨁⨁◯◯ Low |  |
| **Strength and power (follow-up: range 4 weeks to 16 weeks; assessed with: RM test)** | | | | | | | | | | | | |
| 3 | randomized trials | not serious | not serious | not serious | very serious^d^ | none | 55 | 55 | - | SMD **0.29 SD higher** (0.0824 lower to 0.662 higher) | ⨁⨁◯◯ Low |  |

**CI:** confidence interval; **SMD:** standardized mean difference

#### Explanations

a. Equilibrium outcome: variations in effect estimates; 95% CI overlap; Statistically significant heterogeneity; I^2^ (inconsistency) 80.89%

b. Regarding the imprecision of the balance outcome, the N of studies is insufficient and the data do not show significant effects, with the confidence interval crossing the no-effect line.

c. Regarding the agility outcome, the data show a small inconsistency in the variation of effect estimates, a significant heterogeneity, with I^2^ of 62.73%.

d. Regarding the inaccuracy, the N of studies is insufficient and the data, despite appearing to show relevant effects, 95%Cl falls into a range that may not present relevant effects.

e. Variation in effect estimates

f. Insufficient N across studies
